# Authors’ reply

**Published:** 2010

**Authors:** Frederico Castelo Moura, Mário L R Monteiro

**Affiliations:** Neuro-Ophthalmology Service, University of São Paulo Medical School, São Paulo, Brazil

Dear Editor,

We thank Ramchandani *et al*.[1] for the interest in our article[2] and for the opportunity to clarify some issues that were raised.

We completely agree that careful history and clinical examination including visual field testing is sufficient to make the diagnosis after excluding other causes with neuroimaging. Other tests such as optical coherence tomogram (OCT) and even visual evoked potential (VEP) are not necessary for diagnosis in such cases as well as in most visual pathways abnormalities. However, establishing that optic nerve fibers are still intact or permanently damaged is of great interest and has been the subject of extensive research in recent years. While visual acuity and field measurements indicate the presence of optic nerve dysfunction, quantification of axonal loss is interesting information that helps in predicting whether visual recovery is possible or unlikely to occur. Of course we can also grossly estimate axonal loss just by looking carefully at the retinal nerve fiber layer or even at the optic disc color. However, OCT provides an objective way of quantifying axonal loss not only in glaucoma but also in many other anterior optic pathway conditions.[3,4] This is the whole point of evaluating the performance of OCT in different optic neuropathies. In some, structure-function relationship between fields and axonal loss seems to be adequate[5,6] and OCT helps in predicting visual recovery after treatment.[7] In many others its performance is yet not well understood.

The purpose of our paper was to bring to the reader’s attention the possible confusion that can occur when one uses OCT in tobacco-alcohol-induced toxic optic neuropathy. While the first two cases showed the expected pattern of axonal loss in the temporal side of the disc, Case 3 surprisingly did not demonstrate it. It is possible that the finding in Case 3 indicates a lack of sensitivity of OCT. We believe that it indicates that the development of retinal nerve fiber layer loss in this condition may take longer than we previously expected based on our experience with other conditions demonstrating retinal nerve fiber layer loss in the temporal side of the optic disc.[3] We believe it could also indicate that possible axonal loss may have been counterbalanced by nerve fiber edema (possible due to axoplasmic flow stasis). It is therefore important to emphasize that such a pattern can occur in order to avoid diagnostic confusion.

Although we understand that VEP may be helpful in many conditions, we agree that it is not specific and do not believe it is necessary for the diagnosis. We did not use it in any of these cases. We do not believe that the presence of edema should have any correspondence with arcuate scotomas since it is hard to know exactly what the enlargement of the retinal nerve fiber layer signifies.

In conclusion, our study had the purpose of investigating the possible use of OCT in tobacco-alcohol-induced toxic optic neuropathy to quantify axonal loss and to emphasize that if may fail in that regard as documented by one of our cases. We thank for the important observations and for the opportunity to further discuss the subject.
